# Determinants of Advance Directive Completion Among Older Chinese Americans

**DOI:** 10.1093/geroni/igaa057.1526

**Published:** 2020-12-16

**Authors:** Kaipeng Wang, Bei Wu, Fei Sun, Dexia Kong, Yanqin Liu

**Affiliations:** 1 University of Denver, Denver, Colorado, United States; 2 New York University, New York, New York, United States; 3 Michigan State University, East Lansing, Michigan, United States; 4 Rutgers University, New Brunswick, New Jersey, United States; 5 Arizona State University, Tempe, Arizona, United States

## Abstract

Advance Directive (AD) allows older adults to communicate preferred care at the end of life. Numerous studies reported that ethnic minorities were less likely to complete AD then non-Hispanic Whites. However, determinants of AD completion among older Chinese Americans remain unknown. The present study aims to address this knowledge gap. Data came from a survey of 439 Chinese Americans aged from 51 to 103 living in two metropolitan areas in 2018. Participants’ average year was 75 (SD=9.37). About 63% were women and 93% were born outside the US. Approximately 14% of participants completed an AD. Guided by the Andersen’s Service Use Model, we used logistic regression to examine determinants of AD completion. Results show that older age (OR = 1.06, p < 0.01), being employed (OR = 2.63, p < 0.05), acculturation (OR = 2.09, p < 0.001), having US citizenship (OR = 3.57, p < 0.01), and expectation of intergenerational support (OR = 1.84, p < 0.05), were positively associated with AD completion. Physical and mental health needs were not significantly associated with AD completion. This is among the first studies focusing on AD completion among Chinese Americans, one of the fastest growing older minority populations in the US. Findings highlight the influence of socioeconomic and cultural factors on AD completion and illustrates the importance of developing culturally sensitive interventions to promote end-of-life care decision-making among older Chinese Americans.

